# Microplastics Released from Disposable Food-Handling Gloves: Role of Material Type and Food Simulant

**DOI:** 10.3390/ma19102045

**Published:** 2026-05-14

**Authors:** Qifeng Gao, Zixuan Li, Hongyu Liu, Chaonan Zhang

**Affiliations:** College of Life and Environmental Science, Shaoxing University, Shaoxing 312000, China

**Keywords:** microplastics, disposable gloves, polyethylene, thermoplastic elastomer, polylactic acid, oil

## Abstract

The rapid expansion of the takeaway food industry has led to the widespread use of disposable gloves as food-contact materials, which may release microplastics (MPs) during use, posing potential risks to human health and the environment. This study investigated the release of MPs from three common types of disposable food-handling gloves—polyethylene (PE), thermoplastic elastomer (TPE), and polylactic acid (PLA)—into water and edible oil as food simulants. The results indicated that the oil-containing system significantly promoted the release of MPs, with release levels generally 2 to 3 times higher than those in the water environment. Among the materials, PE gloves released the highest amount of MPs in oil, reaching 3183.33 ± 500.83 items/m^2^, while TPE gloves released the lowest amount in water, only 183.33 ± 28.87 items/m^2^. Morphologically, the released MPs were predominantly fibrous, with a notable presence of granular MPs from PE gloves in the oil environment. Surface characterization by Raman spectroscopy and scanning electron microscopy provided additional observations that were broadly consistent with the release patterns. In conclusion, this study highlights the importance of material selection and usage conditions in mitigating MP contamination from disposable food-handling gloves.

## 1. Introduction

Plastics have become an integral part of human life. In recent years, microplastics (MPs), defined as synthetic particulate or heavily modified natural polymers with a particle size <5 mm, have attracted widespread attention as an emerging environmental pollutant [1]. The rapid expansion of the takeaway food industry has significantly increased the consumption of disposable plastic food-contact materials, especially those that come into direct contact with food. More than one-third of global plastic production is used for single-use packaging, which is often improperly disposed of, eventually entering the environment and degrading into MPs [2]. To date, MPs have been widely distributed in marine, freshwater, soil, and even atmospheric environments [3]. Humans may ingest an average of 0.1~5 g of MPs per week through various exposure pathways, posing potential threats to ecosystems and human health [4,5]. Therefore, quantifying the release potential of MPs from such direct-contact items is essential for accurately managing their environmental and health risks.

Food-contact materials can release MPs due to mechanical stress, thermal effects, or chemical swelling during use, and these particles may enter the human body through food ingestion [6]. Studies have shown that steeping a single plastic teabag at 95 °C could release approximately 11.6 billion MPs and 3.1 billion nanoparticles (NPs) into a beverage [7]. Single-use nylon bags and polyethylene (PE) hot drink cups released NPs at densities > 10^12^ particles/L [8], and plastic takeaway containers released 3~29 items/container MPs under hot-filling or mechanical shaking conditions [9]. Disposable gloves, which serve as a direct barrier between hands and food, are typically made of materials such as PE, thermoplastic elastomer (TPE), and polylactic acid (PLA). These plastics gloves are more susceptible to surface wear or degradation during grasping, friction, and contact with oils and water, making them a potential source of MP release. Recent computational approaches, such as reactive molecular dynamics (ReaxFF-MD) simulations, have provided atomic-scale insights into polymer degradation mechanisms and surface interactions, complementing experimental investigations [10,11]. Although the present work is experimentally focused, these computational perspectives help to rationalize material-dependent microplastic release behaviors, such as the contrasting responses of PE and PLA to thermal and solvent exposure.

While existing research has explained the MP release mechanisms of medical disposable gloves in detail [12], studies on the MP release mechanisms of takeaway disposable gloves in daily scenarios remain scarce. This study selected three common takeaway gloves (PE, TPE, PLA), simulated their use in water and oil environments, and systematically investigated the abundance, morphology, and spectral characteristics of released MPs [13,14]. Therefore, this study aims to (i) quantify and compare the release of MPs from PE, TPE, and PLA gloves into water and edible oil; (ii) characterize the morphology and chemical nature of the released MPs; and (iii) elucidate the release mechanisms by systematically analyzing the effects of medium type (water vs. oil) and material properties on MPs release; (iv) reveal medium-induced surface alterations and material-specific degradation behaviors through scanning electron microscopy (SEM) and Raman spectroscopy; and (v) provide crucial data for risk assessment of plastic food-contact articles and inform the selection of safer materials. These findings are expected to fill the current research gap on MP release from disposable takeaway gloves in common daily use scenarios, and provide a scientific basis for formulating relevant material selection standards and environmental risk management strategies for disposable food-contact plastic products.

## 2. Materials and Methods

### 2.1. Sample Selection and Treatment

**Figure 1:**
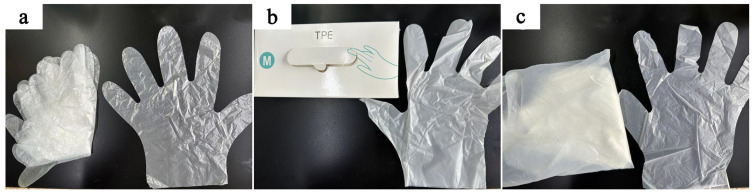
The glove samples selected for this experiment. (**a**) PE glove, (**b**) TPE glove, and (**c**) PLA glove.

The samples used in this experiment were PE gloves (Thickness = 12.70 ± 0.80 μm), TPE gloves (Thickness = 15.95 ± 0.46 μm), and PLA gloves (material: PLA + Polybutylene Adipate Terephthalate + cornstarch, Thickness = 15.63 ± 0.80 μm), all of which were purchased from a local supermarket (Figure 1). The experiment included six experimental groups and two control groups: water-PE, water-TPE, water-PLA, oil-PE, oil-TPE, oil-PLA, water-control, and oil-control. Each experiment was performed in triplicate, and for each repetition, different gloves from the same package were used. The glove samples were cut into 5 cm × 5 cm squares (eight pieces per group) and rinsed three times with distilled water to remove surface contaminants [12]. The cleaned samples were evenly placed into two 100 mL glass beakers. One beaker was filled with 80 mL of distilled water to simulate the water environment, while the other was filled with 20 mL of edible oil (Luhua 5S pressed first-grade peanut oil, Luhua Group, Laiyang, China) and 60 mL of distilled water to simulate the oil environment, as oil-containing media have been shown to significantly enhance microplastic release from plastic materials [15]. Both beakers were placed in a constant-temperature water bath at 40 °C for 2 h. Two additional glass beakers without samples were treated similarly to simulate the water and oil environments as controls. After treatment, the sample liquids were poured into a vacuum filtration pump. The samples and glass beakers were rinsed three times with distilled water, and the rinsate was also filtered. Filtration was performed using 0.22 μm organic filter membranes (Tianjin Jinteng Experimental Equipment Co., Ltd., Tianjin, China), a common pore size for microplastic separation [16]. After filtration, the membranes which contained MPs were placed in petri dishes and air-dried at room temperature.

### 2.2. Quantitative Analysis of Glove Leachates

The filter membranes were observed under a stereomicroscope (SMZ1000, Nikon, Tokyo, Japan) to examine the color and shape of MPs. MPs were counted, classified, and statistically analyzed to calculate their abundance. The MP abundance was calculated as: (number of MP particles per filter membrane)/(sample surface area). The surface area of each glove piece was calculated as twice the geometric area (both sides) because both surfaces were in contact with the food simulant. For a 5 cm × 5 cm square, the total surface area per piece was 2 × (0.05 m × 0.05 m) = 0.005 m^2^. The edge area (thickness × perimeter ≈ 16 μm × 0.2 m = 3.2 × 10^−6^ m^2^) was negligible (<0.1% of total area) and therefore omitted.

### 2.3. Sample Identification and Quantification

A high-resolution confocal Raman microscope (XploRA PLUS, HORIBA France SAS, Palaiseau, France) with the accompanying spectroscopy suite software (LabSpec 6) was used to obtain Raman spectra of the samples before and after treatment. Spectra were acquired under 20× magnification with an excitation wavelength of 532 nm, laser power at 1% on the sample, a spectral grating of 600 gr/mm, a slit width of 100 μm, and a pinhole of 300 μm. The detected spectral range was 100~4000 cm^−1^.

Prior to SEM observation, the samples were sputter-coated with a thin layer of gold to enhance conductivity. This was performed using a sputter coater (MSP-1S, Shinkuu, Tokyo, Japan) at a current of 20 mA for a duration of 60 s under an argon atmosphere. The retained samples were observed using a scanning electron microscope (SU3800, Hitachi, Tokyo, Japan) to capture surface damage at magnifications of 1000× or higher.

### 2.4. Quality Assurance and Control

To minimize external contamination, laboratory personnel wore medical latex gloves throughout the experiment, as well as clean cotton lab coats to reduce contamination from synthetic fibers. All experimental equipment was rinsed with ultrapure water, and glassware was dried after washing. A fume hood and air-treatment devices were used to reduce airborne MP contamination during sampling, sample preparation, and analysis [17]. During filtration, the vacuum filtration pump and filter funnels were covered with clean aluminum foil or glass domes to prevent deposition of airborne particles. To ensure counting reliability, all filter membranes were counted by the same trained operator. The same operator selected a random sample from each group again after one week for re-counting, yielding a relative standard deviation (RSD) of <10% for total particle counts. Procedural blank controls were also set up alongside all experiments to detect contamination introduced during sample processing. For each batch of experiments, one blank control was processed following the exact same filtration and drying procedure without any test sample, and the number of MPs found in blank controls was subtracted from the corresponding sample counts before statistical calculation.

### 2.5. Statistical Analyses

Statistical analyses were performed with IBM SPSS Statistics v19.0 by the two-way analysis of variance (two-way ANOVA) after verification of the normality and variance. A significance level of *p* < 0.05 indicated statistical significance. The expression profiles among the four groups were analyzed by two-way ANOVA with Tukey’s test. Data visualization was carried out using Origin 2024 and GraphPad Prism 9.0.0 (San Diego, CA, USA).

## 3. Results

### 3.1. The Observation Results of the Filter Paper by Stereomicroscope

**Figure 2:**
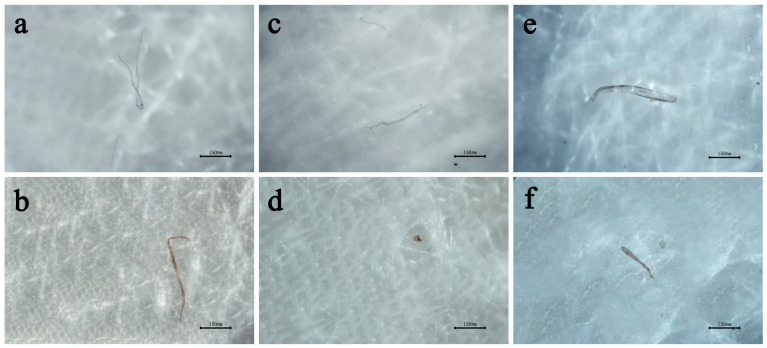
MPs reference image collected from the filter membrane. (**a**) Water-PE group. (**b**) Oil-PE group. (**c**) Water-TPE group. (**d**) Oil-TPE group. (**e**) Water-PLA group. (**f**) Oil-PLA group.

Observation of the control group filter papers revealed an almost complete absence of MPs in both water and oil controls, thereby ruling out external contamination as a significant factor affecting the experimental results. Comparing the results from the experimental groups, it was found that the MPs released by PE, TPE, and PLA gloves in either water or oil were predominantly fibrous in morphology (Figure 2). In the aqueous environment, the microplastics appeared as transparent fibers, whereas in the oil environment, most MPs were stained a brownish-yellow color by the cooking oil. Filmy and granular MPs constituted minimal proportions across all groups, with the notable exception of the oil-treated PE gloves, which released a substantial quantity of fine, white granular MPs.

### 3.2. MPs Abundance and Morphology

**Figure 3:**
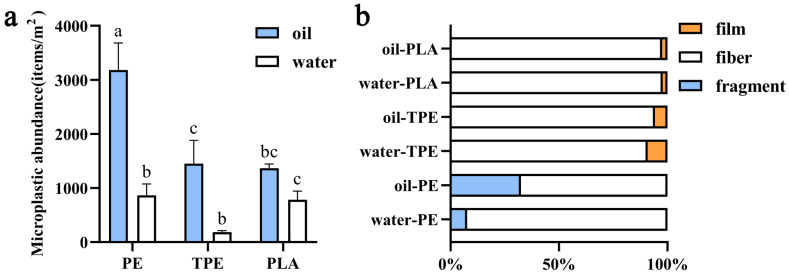
(**a**) Bar charts of the abundance of microplastics in each group. (**b**) The proportion of microplastic morphology. Different letters are significant differences (*p* < 0.05).

Each filter paper corresponded to four glove fragments, and the calculated surface area of the samples was 0.02 m^2^. The calculated abundances of MPs were 866.67 ± 208.17 items/m^2^ in the water-PE group, 3183.33 ± 500.83 items/m^2^ in the oil-PE group, 183.33 ± 28.87 items/m^2^ in the water-TPE group, 1450.00 ± 433.01 items/m^2^ in the oil-TPE group, 783.33 ± 125.83 items/m^2^ in the water-PLA group and 1366.67 ± 76.38 items/m^2^ in the oil-PLA group. Meanwhile, the content of microplastics in the water control group was 16.67 ± 23.57 items/m^2^, and in the oil control group it was 50.00 ± 40.82 items/m^2^. These amounts were extremely low and could be considered negligible compared to the treatment group. The amount of microplastics released by PE and TPE gloves in the oil treatment group was significantly higher than that in the water treatment group (*p* < 0.05), and the amount of microplastics released by PE gloves in the oil treatment group was significantly higher than that in all other groups (*p* < 0.05, Figure 3a). The results of the two-way ANOVA were presented in Table 1.

The MPs released from all sample groups exhibited diverse morphologies, with white being the predominant color (Figure 3b). Overall, fibrous MPs predominated, while only small quantities of filmy MPs were observed in the TPE and PLA groups. White granular MPs were present in PE samples, with their proportion increasing significantly in the oil environment. Filmy MPs accounted for 9.09% in TPE samples immersed in water, and were less frequent (5.75%) in the oil environment. Only minor amounts of filmy MPs were detected in PLA samples.

### 3.3. SEM Results

**Figure 4:**
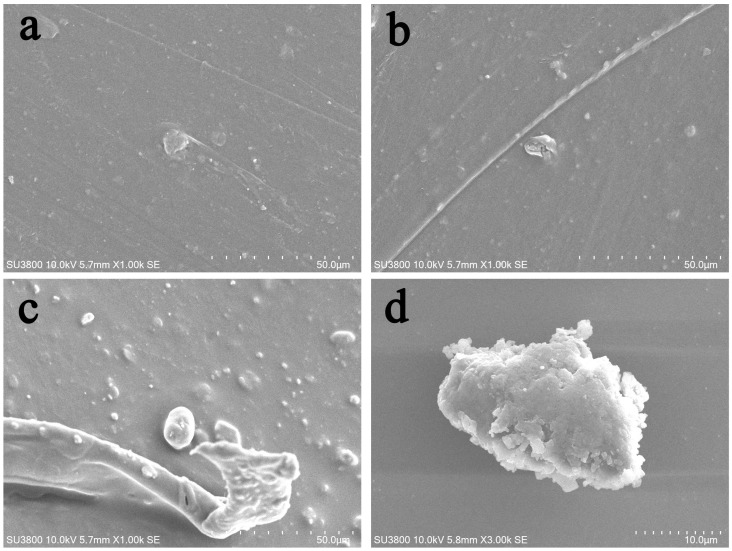
SEM images. (**a**) Untreated PE gloves. (**b**) PE gloves treated by the water group. (**c**) PE gloves treated by the oil group. (**d**) Presents the magnified images of (**c**).

Based on the results in Figure 3, the PE gloves with the highest MPs abundance were selected for SEM observation. The untreated PE glove showed a largely intact surface with minimal damage (Figure 4a). The water-treated PE glove showed obvious but limited damages on the surface (Figure 4b). The oil-treated PE glove showed extensive damage, with granular and fibrous substances clearly peeling off (Figure 4c). The selected granular substances in the oil-treated PE glove were presented through an enlarged display (Figure 4d). In conclusion, compared with water treatment, oil treatment caused significant damage to the surface of PE gloves and a large amount of granular and fibrous MPs peeling off.

### 3.4. Raman Images

**Figure 5:**
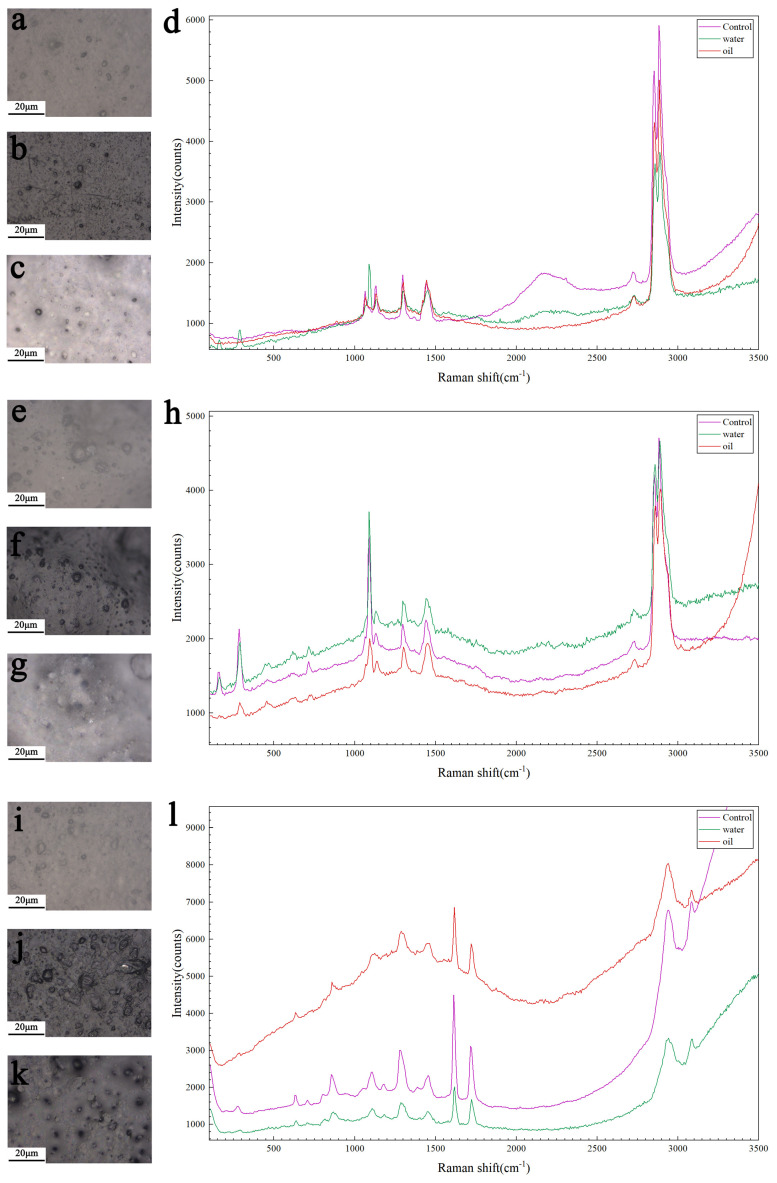
Photo images (**a**–**c**,**e**–**g**,**i**–**k**), and typical Raman spectra (**d**,**h**,**l**) collected from gloves before and after processing. (**a**–**c**) present the surface images of PE gloves during Raman scanning for the control, water-treated, and oil-treated groups, respectively, (**e**–**g**) show those of TPE gloves under the corresponding conditions, and (**i**–**k**) display those of PLA gloves. (**d**) compares the Raman spectra of PE gloves between the control and treatment groups, (**h**) compares those of TPE gloves, and (**l**) compares those of PLA gloves.

PE gloves exhibit distinct characteristic peaks at 1100 cm^−1^, 1340 cm^−1^, 1450 cm^−1^, and 2880 cm^−1^. TPE gloves show clear characteristic peaks at 300 cm^−1^, 1200 cm^−1^, 1340 cm^−1^, 1450 cm^−1^, and 2880 cm^−1^. PLA gloves display pronounced characteristic peaks at 650 cm^−1^, 850 cm^−1^, 1300 cm^−1^, 1450 cm^−1^, 1630 cm^−1^, 1750 cm^−1^, and 2880 cm^−1^. Some of these characteristic peak bands are similar to those reported by Luo et al. [18], and are assignable to polymeric materials [19]. The peak at 2880 cm^−1^ is the most intense across all glove groups (Figure 5).

The characteristic Raman peaks of PE gloves showed a decrease in intensity after both water and oil treatment, with a more pronounced reduction observed in the water-treated sample (Figure 5d). In contrast, TPE gloves after water treatment exhibited an increase in the overall spectral intensity, particularly in the fluorescence background (Figure 5h). PLA gloves after oil treatment showed a significant increase in peak intensity, accompanied by an elevated spectral baseline (Figure 5l).

## 4. Discussion

### 4.1. Oil Medium Substantially Promotes MP Release

The results of this study indicated that the abundance of MPs released from PE, TPE, and PLA gloves was significantly higher in the oil environment than in the water environment, with an overall release level approximately 3.3 times higher. This significant difference may be primarily related to the physicochemical properties of edible oil [20]. As a nonpolar organic solvent, edible oil could exert a swelling and softening effect on nonpolar polymers such as PE. Oil molecules can penetrate between polymer chains, weakening intermolecular forces and making plastic fragments or particles more prone to detachment under mechanical or thermal stress [21]. During the constant-temperature heating and subsequent treatment in this experiment, this swelling effect might make the material surface more susceptible to peeling under minor mechanical stress, forming filmy, granular, or fibrous MPs. This phenomenon is consistent with findings by Huang et al., who reported that fatty foods enhance MP release from plastic containers, possibly due to lipid-induced polymer swelling [22]. In contrast, water, as a polar molecule, has much lower wetting and swelling capacity for nonpolar plastics, which could explain less structural damage and correspondingly lower MP release. Notably, different materials exhibited distinct responses to the oil environment. Although TPE and PLA exhibited significant increase in MP release in oil, their release levels remained far below those of PE, suggesting that the material’s physicochemical structure and oil resistance are key intrinsic factors determining release behavior. The extent of release varied among materials, with PE showing the highest sensitivity to oil, likely due to its simple hydrocarbon structure and low intermolecular cohesion [23]. Therefore, disposable gloves may become a more significant source of MP pollution when handling high-fat foods.

### 4.2. Polymer Type Governs Release Magnitude

This study revealed that gloves made of different materials exhibit significant differences in MP release capacity in the same medium. In the water treatment group, the PE gloves released the largest number of microplastic particles, while the TPE gloves released the smallest number of microplastic particles—a difference of up to 4.7-fold, which appears to be related to the material’s physical properties and chemical structure. PE is a nonpolar polymer with a simple molecular chain structure, relatively high crystallinity, but likely weak intermolecular forces, making it particularly sensitive to oil swelling and potentially prone to releasing large amounts of MPs [24]. TPE, as a thermoplastic elastomer, has good structural stability and some resistance to medium erosion [25]. PLA, as a bio-based material, may degrade or delaminate under medium interaction due to structural inhomogeneity after blending with PBAT and starch, which could lead to MP release [26].

The material-dependent release behavior highlights the potential role of polymer composition and structure in determining release potential. TPE, comprising rigid and soft segments, likely exhibits greater structural integrity and resistance to medium penetration, which may explain its lower MP release in both water and oil [27]. PLA, although biodegradable, is a semi-crystalline polyester that can undergo hydrolysis in aqueous environments [28]. However, the observed increase in MP release from PLA in oil suggested that oil might act as a plasticizer or induce surface rearrangement, promoting the detachment of amorphous regions or blended components. Such behavior has been noted in composite biodegradable films exposed to lipid-rich media [26].

### 4.3. Morphological Signatures Reflect Distinct Release Mechanisms

The morphological characteristics of MPs can reflect their release mechanisms. In this study, fibrous MPs were the dominant morphology, especially in the PE and PLA groups, which could be related to the fiber residues during material processing or mechanical friction during use. In the oil environment, the PE group released a large number of granular MPs, which is consistent with the idea that oil swelling caused surface peeling. The low proportion of filmy MPs in TPE and PLA suggested relatively stable structures that were less prone to layered peeling [29]. The significant increase in granular MPs from PE in oil further supported the destructive effect of oil on its surface structure.

The predominance of fibrous MPs across all samples suggested that mechanical abrasion during glove manufacturing or use could generate and release polymer fibrils. Similar fibrous MP release has been reported for synthetic textiles and fragmented plastic products [24]. The presence of granular MPs in oil-treated PE samples points to a different release mechanism—likely surface erosion and chunk detachment due to oil-induced swelling and softening, which was supported by SEM images showing pronounced surface damage and granular debris on oil-exposed PE gloves (Figure 4c). The low proportion of filmy MPs in TPE and PLA implied that these materials are less susceptible to laminar peeling, possibly due to better interlayer adhesion or higher elasticity. These morphological patterns provided visual evidence of how different degradation mechanisms—fibrillation, erosion, and delamination—operate under varying environmental conditions.

### 4.4. Raman Spectral Shifts Indicate Material-Specific Surface Alterations

After treatment with water and oil media, the Raman characteristic peaks of PE gloves (especially in the 2800~3000 cm^−1^ range) weaken significantly, with a more pronounced reduction in the water group. One plausible interpretation was physical peeling and mass loss from the sample surface. Under the swelling and softening effects of water and oil, surface polymers might detach as MPs, reducing the total amount of Raman-signal-generating material per unit area, thus lowering spectral intensity [30]. The greater reduction observed in the water group might be attributed to the detachment of larger fragments, leading to more pronounced overall signal attenuation. Notably, the absolute release amount was lower than that in the oil group, suggesting differences in particle number or size distribution between the two groups. However, without independent mass loss or swelling measurements, this remains tentative.

The spectral changes in TPE gloves are more complex. After oil treatment, Raman peaks intensity decreased, which is reminiscent of the behavior observed for PE, and could be due to oil swelling of nonpolar components in TPE, causing surface polymer detachment and mass loss. However, after water treatment, the Raman peaks appeared enhanced. This does not indicate an increase in material but is more likely due to the strong fluorescence background. TPE typically consists of hard and soft segments and certain components (such as polyolefin soft segments or additives) may undergo slight migration or oxidation under water and heat, potentially producing fluorescent molecules [31]. When excited by laser, these molecules could have generated a broad, continuous, and intense fluorescence background that elevated the baseline, making the Raman spectrum appear stronger [32]. Such an artifact could obscure the true Raman signal. Simultaneously, water might erode the material surface, altering light-scattering properties and indirectly affecting signal collection efficiency. Therefore, the enhancement of the Raman signal in water-treated TPE may primarily reflect changes in the surface chemical state and fluorescence interference, rather than higher stability—consistent with the lowest MP release indicating that such changes occur at the molecular scale or involve particle release without forming observable MPs. The weakening of the Raman signal in oil-treated TPE reflected substantial surface polymer detachment, consistent with the significantly higher MP abundance in oil-treated TPE compared to that in water-treated TPE. We note, however, that without complementary fluorescence control experiments or surface chemistry analysis, the fluorescence interpretation remains a hypothesis.

PLA gloves exhibited the most distinctive spectral changes. After water treatment, the Raman peaks weakened as expected, which could be attributed to slight hydrolysis of PLA ester bonds at 40 °C, if it occurs. This hydrolysis led to polymer chain breakage and dissolution of degradation products, resulting in mass loss and a corresponding reduction in signal intensity. In contrast, after oil treatment, the Raman peaks significantly strengthened. PLA is a semi-crystalline polymer that exists in an amorphous or low-crystallinity state after processing. In this study, we hypothesize that edible oil at 40 °C may act as a thermal medium, perhaps exerting an annealing-like effect on PLA. This mild thermal treatment could promote molecular chain mobility and rearrangement, thereby increasing crystallinity in the near-surface region. Higher crystallinity could enhance Raman scattering efficiency due to the more ordered arrangement of polymer chains [33]. Additionally, the oil might selectively dissolve amorphous components or small-molecule additives from the PLA/PBAT/cornstarch blend, increasing surface roughness and creating more laser scattering points, which might further amplify the Raman signal [34]. Therefore, the enhanced Raman signal in oil-treated PLA could reflect reconstruction of the surface microstructure and physical state—namely crystallization and roughening—rather than the absence of MPs release. In fact, MP release likely occurred during this surface reconstruction process, with the detached portions being the less stable amorphous regions. Nevertheless, these interpretations are speculative; confirmation would require crystallinity measurements and surface chemical imaging.

### 4.5. Implications for the Environment and Human Health

This study demonstrated that disposable takeaway gloves would release MPs in both water and edible oil environments, with a significantly higher release in oil. This means that during daily use, especially when handling high-fat foods, gloves may become a direct source of MP release. These findings suggest that handling high-fat foods could be a scenario where MP release from gloves occurs. However, the actual transfer of released particles from gloves to food, and subsequently to the human body, depends on factors not evaluated here. These released MPs may enter the human body through dietary intake of contaminated food, as emerging evidence demonstrates that MPs and NPs migrate from food-contact materials into foodstuffs during normal use and subsequently bioaccumulate in human tissues [35]. Meanwhile, MPs released from disposable gloves during washing and disposal can enter aquatic environments via wastewater effluents, where treatment plants often exhibit limited removal efficiency, contributing to the growing burden of microplastic pollution in freshwater and marine ecosystems [36]. Moreover, fibrous MPs, which dominated the release profiles in the present study, have been considered to exhibit prolonged residence time in the gastrointestinal tract and greater potential to penetrate the mucus layer, leading to localized inflammatory responses [37]. Qiao demonstrated that microplastic fibers accumulated in the zebrafish gut at significantly higher levels (8.0 μg/mg) than fragments and beads, and caused more severe mucosal damage, increased intestinal permeability, and inflammation compared to other morphologies [38].

From a food safety perspective, standards and regulations for MP release from food-contact materials remain underdeveloped. The quantitative data provided in this study—on MP abundance, morphology, and material-dependent release patterns—may serve as a reference for future risk assessments. It is recommended to prioritize materials with better oil resistance in high-fat food-contact scenarios and strengthen environmental risk assessment throughout the lifecycle of disposable plastic products. Any recommendations on material selection should be considered preliminary, as they are based on controlled laboratory conditions that do not fully capture real world use.

### 4.6. Limitations and Future Perspectives

The present experiments were conducted at 40 °C, a moderate and conservative thermal condition relevant to warm food handling and regulatory migration testing. However, real-world scenarios may involve higher temperatures (60–80 °C) during initial packaging of freshly cooked foods, as well as mechanical stress, temperature fluctuations, and prolonged use. Given that MP release from polypropylene food containers has been reported to increase by up to two orders of magnitude when water temperature rises from 25 °C to 95 °C, the present results obtained at 40 °C may underestimate real-world exposure levels during high-temperature food handling scenarios [39]. Our results demonstrate that disposable takeaway gloves can release substantial quantities of MPs, particularly in oil-rich environments, with release levels significantly influenced by polymer type. PE proved most prone to fragmentation. These findings highlight gloves as a notable yet variable source of MP pollution in food-contact contexts. Nevertheless, this work has certain limitations. First, particles smaller than 1 μm were not characterized by the optical counting method. Therefore, our reported particle counts (items/m^2^) primarily reflect the >1 μm fraction. Second, recovery efficiency tests using standard microplastics were not conducted. However, procedural blanks confirmed negligible external contamination. Third, inter-operator variability was not systematically assessed, though intra-operator repeatability was acceptable (RSD < 10%). Fourth, the experimental conditions (40 °C) did not fully capture the mechanical stress, temperature fluctuations, prolonged use, and higher temperatures encountered in real-world scenarios. Furthermore, while elevated temperatures can promote MP release, they may also induce material deformation or melting (especially for PLA), as well as concurrent changes in crystallinity and hydrolytic degradation, collectively complicating mechanistic interpretation [40].

To better assess environmental and health risks, future research should systematically investigate temperature-dependent MP release across a gradient to capture real-world thermal variability and establish temperature–release relationships. Additionally, subsequent studies should (i) employ more realistic use simulations including mechanical stress and temperature gradients; (ii) characterize released nanoparticles using techniques such as nanoparticle tracking analysis or field-flow fractionation; (iii) investigate the toxicological effects of glove-derived MPs on cellular and organismal models; and (iv) explore the influence of oil composition and food matrices on MP release. Establishing science-based guidelines for glove production, usage, and waste management requires coordinated efforts across research, industry, and policy sectors.

## 5. Conclusions

This study examined the release of MPs from three commercially available disposable food-handling glove products, respectively labeled as PE, TPE, and PLA, under simulated water and edible oil environments. Oil significantly promoted MP release, yielding abundances 2–3 times higher than in water. PE gloves exhibited the highest release in oil (3183.33 ± 500.83 items/m^2^), whereas TPE gloves showed the lowest in water (183.33 ± 28.87 items/m^2^). Released MPs were predominantly fibrous, with a notable increase in granular particles from PE in oil. SEM imaging of PE gloves confirmed extensive surface damage and particle detachment after oil exposure. Raman spectral changes—including reduced PE peak intensities, fluorescent interference in water-treated TPE, and enhanced PLA signals post-oil treatment—were broadly consistent with the observed release patterns. These findings highlight that both polymer type and contact medium critically govern MP release, and that TPE gloves released substantially fewer MPs than PE under the tested conditions. Future research should incorporate more realistic use conditions, including mechanical stress, and focus on the release and toxicology of sub-micrometer particles based on the current preliminary findings.

## Figures and Tables

**Table 1:** Results of the two-way ANOVA.

Source of Variation	SS	df	MS	F (DFn, DFd)	*p*-Value
Row Factor	4,858,611	2	2,429,306	F (2, 12) = 28.35	0.0009
Column Factor	8,680,556	1	8,680,556	F (1, 12) = 101.3	<0.0001
Interaction	2,286,944	2	1,143,472	F (2, 12) = 13.34	<0.0001
Residual	1,028,333	12	85,694		

Note: The row factor represented the differences among materials (PE, TPE, PLA), and the column factors represented the differences among medium (water, oil). SS: Sum-of-squares. MS: Mean squares. Each mean square value is computed by dividing a sum-of-squares value by the corresponding degrees of freedom. df: Degrees of freedom. F: F ratio. Each F ratio is computed by dividing the MS value by another MS value.

## Data Availability

The original contributions presented in this study are included in the article. Further inquiries can be directed to the corresponding author.
